# Association between *NME8* Locus Polymorphism and Cognitive Decline, Cerebrospinal Fluid and Neuroimaging Biomarkers in Alzheimer's Disease

**DOI:** 10.1371/journal.pone.0114777

**Published:** 2014-12-08

**Authors:** Ying Liu, Jin-Tai Yu, Hui-Fu Wang, Xiao-Ke Hao, Yu-Fen Yang, Teng Jiang, Xi-Chen Zhu, Lei Cao, Dao-Qiang Zhang, Lan Tan

**Affiliations:** 1 Department of Neurology, Dalian Medical University, Qingdao Municipal Hospital, Qingdao, China; 2 Department of Neurology, Qingdao Municipal Hospital, School of Medicine, Qingdao University, China; 3 Department of Neurology, Qingdao Municipal Hospital, Nanjing Medical University, Nanjing, China; 4 Department of Computer Science and Engineering, Nanjing University of Aeronautics and Astronautics, Nanjing, China; 5 Air Beijing Dongjiao Lane Community Health, Beijing, China; 6 Department of Neurology, Nanjing First Hospital, Nanjing Medical University, Nanjing, China; The Florey Institute of Neuroscience and Mental Health, Australia

## Abstract

Recently, a large meta-analysis of five genome wide association studies (GWAS) identified a novel locus (rs2718058) adjacent to *NME8* that played a preventive role in Alzheimer's disease (AD). However, this link between the single nucleotide polymorphism (SNP) rs2718058 and the pathology of AD have not been mentioned yet. Therefore, this study assessed the strength of association between the *NME8* rs2718058 genotypes and AD-related measures including the cerebrospinal fluid (CSF) amyloid beta, tau, P-tau concentrations, neuroimaging biomarkers and cognitive performance, in a large cohort from Alzheimer's Disease Neuroimaging Initiative (ADNI) database. We used information of a total of 719 individuals, including 211 normal cognition (NC), 346 mild cognitive impairment (MCI) and 162 AD. Although we didn't observe a positive relationship between rs2718058 and AD, it was significantly associated with several AD related endophenotypes. Among the normal cognitively normal participants, the minor allele G carriers showed significantly associated with higher CDRSB score than A allele carriers (*P* = 0.021). Occipital gyrus atrophy were significantly associated with *NME8* genotype status (*P* = 0.002), with A allele carriers has more atrophy than the minor allele G carriers in AD patients; lateral ventricle (both right and left) cerebral metabolic rate for glucose (CMRgl) were significantly associated with *NME8* genotype (*P*<0.05), with GA genotype had higher metabolism than GG and AA genotypes in MCI group; the atrophic right hippocampus in 18 months is significantly different between the three group, with GG and AA genotypes had more hippocampus atrophy than GA genotypes in the whole group. Together, our results are consistent with the direction of previous research, suggesting that *NME8* rs2718058 appears to play a role in lowering the brain neurodegeneration.

## Introduction

Alzheimer's disease (AD), the most common cause of dementia, is known to be an age dependent neurodegenerative disorder that results in progressive loss of cognitive function and eventually causes death [1], [2]. Epidemiologic studies failed to identify one single cause of AD, although age and positive family history are clearly important risk factors [3]. The most potent risk factor is apolipoprotein ε4 (*APOE* ε4) allele, which had been identified as the most robust susceptibility loci in the late onset AD (LOAD). Since the elucidation of the *APOE* locus in 1993 [4], genome-wide association studies (GWAS) have discovered hundreds of single-nucleotide polymorphisms (SNPs) that are significantly associated with variation in brain structure and function [5]–[8]. To test the role of genetic factors in progress of AD, a growing number of studies are using an intermediate phenotype approach, which utilizes biomarkers, such as structural brain imaging, fluorodeoxyglucose positron emission tomography (PET) measurements of the regional cerebral metabolic rate for glucose (CMRgl), as endpoints in genetic analyses of risk. It helps to understand complex disorders of nervous system, including AD, from genetic determinate to cellular process to the complex interplay of brain structure, function, behavior and cognition.

Recently, a large, two-stage meta-analysis of genome-wide association studies (GWAS) in individuals of European ancestry identified 19 loci, in addition to the *APOE* ε4 loci, reached genome-wide significance in the combined 2 stages, of which 11 are newly associated with AD [9]. Herein, a new discrete identified preventive loci (SNP rs2718058), adjacent to *NME8* (encoding NME/NM23 family member 8), which had never been reported to be associated with AD, was strongly associated with the prevention of AD in both the two stages of the meta-analysis. Of note, little is known about the function of this genetic locus in the progression of AD. The aim of this study was to use the neuroimaging and cognitive phenotypes that have been associated with AD, and test whether the new preventive SNP rs2718058 in *NME8* impacts its phenotypes in a large sample from the Alzheimer's Disease Neuroimaging Initiative (ADNI), which is an NIH-sponsored, multi-site study assessing MRI, PET, biological, clinical and neuropsychological traits to measure the mildly affected AD patients, mild cognitive impairment (MCI) patients, and normal cognition (NC).

## Methods

### Ethics Statement

The ADNI study was approved by the local institutions's Research Ethic Boards (IRBs) of all participating sites. All subjects and if applicable, their legal representatives, gave written informed consent prior to the collection of clinical, genetic and imaging data.

### Subjects and data sources

The data used in the preparation of this paper were obtained from the Alzheimer's Disease Neuroimaging Initiative (ADNI) database (https://ida.loni.usc.edu/). The ADNI was launched in 2003 by the National Institute on Aging (NIA), the National Institute of Biomedical Imaging and Bioengineering (NIBIB), the Food and Drug Administration (FDA), private pharmaceutical companies and non-profit organizations, as a $60 million, 5-year public-private partnership [10]. The primary goal of ADNI has been to test whether serial magnetic resonance imaging (MRI), positron emission tomography (PET), other biological markers, and clinical and neuropsychological assessment can be combined to measure the progression of MCI and AD. Determination of sensitive and specific markers of very early AD progression is intended to aid researchers and clinicians to develop new treatments and monitor their effectiveness, as well as lessen the time and cost of clinical trials.

The Principal Investigator of this initiative is Michael W.Weiner, MD, VA Medical Center and University of California-San Francisco. The initial goal of ADNI was to recruit 800 subjects but ADNI has been followed by ADNI-GO and ADNI-2. The follow up duration of each group is specified in the protocols for ADNI-1, ADNI-2 and ADNI-Go (see www.adni-info.org for up-to-date information). Data for this present analysis were downloaded from the ADNI web site (ADNI-1 data) in November, 2010, and updated in September, 2014. In this paper, only ADNI1 subjects with all detailed clinical information and genotype data for rs2718058 were include, because that there are no more data of rs2718058 available in ADNI-2 and ADNI-Go. This yields a total of 719 subjects including 162 AD, 346 MCI patients and 211 normal healthy older individuals. Table 1 lists the detailed demographics of all these subjects divided to three diagnostic groups (NC, MCI and AD).

**Table 1:** Baseline Demographic, clinical, and neuroimaging characteristics of study participants.

	Means (SD) where given			
Characteristic	NC (n = 211)	MCI (n = 346)	AD (n = 162)	P
Age, year	75.6 (4.9)	74.5(7.3)	75.2(7.3)	0.114
Male sex, No. (%)	114(54)	227(65.6)	83(51.2)	0.8
Education level, year	16.1(2.8)	15.6(3.0)	14.7(3.2)	<0.01
APOE ε4 (0/1/2)	156/51/4	157/148/41	48/82/32	<0.01
CDRSB score∧	0.03(0.13)	1.63(0.95)	4.45(2.03)	<0.01
ADAS11 score°	6.22(2.9)	11.56(4.7)	18.94(7.1)	<0.01
ADAS13 score*	9.54(4.1)	18.72(6.5)	29.23(8.4)	<0.01
MMSE score°	29.0(1.0)	26.9(2.1)	23.1(2.6)	<0.01
RAVLT total score^£^	43.2(9.1)	30.4(9.6)	22.9(7.2)	<0.01
FAQ score^§^	0.14(0.62)	3.97(4.59)	13.3(7.03)	<0.01
Hippocampus, mm^3^**	7259.8(895.4)	6379.9(1069.2)	5602.5(1059.2)	<0.01
Genotype (GG/GA/AA)	35/98/78	53/152/141	19/79/64	0.31
Tau ^#^	68.5(28.1)	101.1(61.8)	88(121.1)	<0.01
Aβ_1-42_^#^	206.7(55.5)	162.4(55.6)	88(143.8)	<0.01
pTau181^#^	24.27(13.6)	35.28(17.9)	41.5(19.5)	<0.01

Abbreviations: NC, normal control; MCI, mild cognitive impairment; AD, Alzheimer's disease; n, number; CDRSB, Clinical Dementia Rating scale sum of boxes; ADAS, Alzheimer's disease Assessment Scale; MMSE, Mini-Mental State Exam; RAVLT, Rey Auditory Verbal Learning Test; FAQ, Functional Activities Questionnaire

∧For CDRSB score, the sample size: NC = 210; °For ADAS11 scores and MMSE scores, the sample size: MCI = 345; *For ADAS13 scores, the sample size: MCI = 342, AD = 158; ^£^For RAVLT total scores, the sample size: NC = 210, MCI = 345, AD = 161; ^§^For FAQ scores, the sample size: MCI = 344; **For Hippocampus volume, the sample size: NC = 193, MCI = 293, AD = 133; ^#^the sample size: NC = 108, MCI = 176, AD = 88

P values for continuous variables are from one-way ANOVA; P values for categorical data are from Spearman's correlation analysis.

### Genetic data

ADNI samples were genotyped using the applied the Human 610-Quad Bead Chip (Illumina, Inc., San Diego, CA) included 620,901 SNP and CNV markers to conduct genotyping [11]. After sample verification and quality control bioinformatics, the genotype data for participants included was uploaded to the ADNI website (http://www.loni.ucla.edu/ADNI). Here we extracted the genetic data of *NME8* (rs2718058) (n = 719) from the PLINK data (Table 1). Detailed sample numbers of each biomarker were shown in Table 2.

**Table 2:** Detailed include numbers of samples in different biomarkers research.

Study	Biomarkers	NC	MCI	AD	Total
**Genotype data**	Total included	211	346	162	719
**Neuropsychological test**	CDRSB	**210**	346	162	718
	ADAS11	211	**345**	162	718
	ADAS13	211	**342**	**158**	711
	MMSE	211	345	162	718
	RAVLT	**210**	345	**161**	716
	FAQ	211	**344**	162	717
**CSF biomarkers**	Tau/Aβ_1-45_/P-tau	**108**	**176**	**88**	372
**MRI**	Regional brain volume	**208**	**343**	**156**	707
	Hippocampus volume	**193**	**293**	**133**	619
**PET**	CMRgl on FDG-PET	**96**	**179**	**85**	360

Abbreviations: NC, normal control; MCI, mild cognitive impairment; AD, Alzheimer's disease; n, number; CDRSB, Clinical Dementia Rating scale sum of boxes; ADAS, Alzheimer's disease Assessment Scale; MMSE, Mini-Mental State Exam; RAVLT, Rey Auditory Verbal Learning Test; FAQ, Functional Activities Questionnaire; CMRgl, the regional cerebral metabolic rate for glucose; PET, fluorodeoxyglucose positron emission tomography.

### CSF biomarker data

Levels of amyloid-beta1-42 (Aβ1-42), tau, and phosphorylated tau (p-tau) were measured from all available CSF samples as described previously [12]. For our analysis between the CSF biomarkers (including Aβ_1-42_, tau, and p-tau_181p_) and the phenotype of rs2719058 SNP, a total of 372 participants (AD = 88, MCI = 176, NC = 108) with genetic and other detailed information were finally include in our analysis from the ADNI website (Table 2).

### Neuroimaging data

These neuroimaging data, including regional volume on MRI, and cerebral metabolic rate for glucose (CMRgl) on FDG-PET were all downloaded from the ADNI dataset. A detailed description of PET image acquisition and processing can be found at http://adni.loni.usc.edu/data-samples/pet/ [13]. Based on the data of the 93 regions of interest (ROIs), we calculated the relationship between *NME8* rs2718058 and AD. We compared the changed values of the hippocampal volume in the five different phases (6 months, 12 months, 18 months, 24 months and 36 months). Totally, there were 707 (AD = 156, MCI = 343, NC = 208) included in the regional brain volume analysis and 360 (AD = 85, MCI = 179, NC = 96) included in the cerebral metabolism analysis at baseline.

### Neuropsychological test

We calculated the cognitive scores of the Clinical Dementia Rating scale sum of (CDRsb), Alzheimer's disease Assessment Scale (ADAS), Mini-Mental State Exam (MMSE), Rey Auditory Verbal Learning Test (RAVLT), and Functional Activities Questionnaire (FAQ) to analysis the cognitive changes of *NME8* gene carriers. We included 719 subjects with genetic and other information at baseline from ADNI-1 dataset, and compared the scores on these scales at baseline and their changed scores after 2 years.

### Statistical Methods

For continuous variables, differences were calculated by one-way analysis of variance (ANOVA); for categorical data, differences were tested by Spearman's correlation analysis. We completed statistics across three diagnostic subgroups (AD, MCI and NC) to test the association of rs2718058 with the disease phenotype respectively. To explore this association further, we conducted a multiple linear regression analysis which consider age, gender, *APOE* ε4 status as covariates in total sample, and calculated the 95% confidence interval (CI) to test the correlation further. All statistical analyses were performed by SPSS 19.0 statistics for IBM. For each positive association, the power was calculated as the percentage of replicates for which the P value was lower than 0.05. Statistical power analyses were performed by PASS11.0. All statistical tests were two-sided, and the statistical significance was defined as P<0.05.

## Results

### Demographics, clinical characters and Neuropsychological test

Demographic characteristics and multiple memory scores for three diagnostic groups (NC, MCI and AD) are presented in Table 1. While baseline age and gender were not significantly different across diagnostic groups, hippocampus volume exhibited significant differences across groups (P<0.01), with hippocampus volume relatively atrophic among AD subjects. Also as expected, the cognitive scores of the six scales (CDRsb, ADAS11, ADAS13, MMSE, RAVLT, FAQ) and *APOE* ε4 allele status differed across groups, including all pairwise group comparisons (P<0.01) (Table 1). As expectedly, significantly more AD were *APOE* ε4+ than NC and E-MCI group (*P*<0.01): 114 of162 AD participants (70.4%) showing one or more ε4 allele relative to 55 of 211 NC participants (26.6%) and 189 of 346 E-MCI participants (54.6%). Furthermore, the volume of hippocampus, the CSF tau, Aβ_1-42_ and pTau181 were all significantly different among three groups, with the AD group has smaller hippocampal volume, lower tau, lower Aβ and higher p-Tau levels in CSF. However, we didn't find significant difference of *NME8* genotype across the three groups (*P* = 0.31).

In the exploration of genotype effect on cognitive function calculated by Neuropsychological test, the rs2718058 was significantly associated with the CDRSB score at baseline in analysis of variance (ANOVA) in the normal participants group, with the CDRSB score in G allele carriers (GG, GA) was marginally significantly higher than AA allele (*P* = 0.050) (S1 Table). We didn't find a significant difference on ADAS-11, ADAS-13, MMSE, RAVLT, and FAQ neither in the total sample nor the three diagnose groups (AD, MCI, NC) (S1 Table). In our analysis, we also calculated the 2-year changes of the six cognitive scales (CDRSB, ADAS-11, ADAS-13, MMSE, RAVLT, and FAQ), we observed a strong significant difference on the changed scores of CDRSB in the NC group (*P* = 0.021), and no remarkable difference on the other scales (S2 Table).

#### 3.2. Three cerebrospinal fluid (CSF) biomarkers analysis

**Figure 1:**
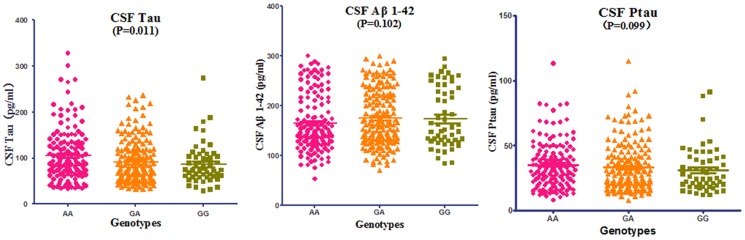
The analysis of three Cerebrospinal fluid (CSF) biomarkers and genotype of NME8 at baseline in total sample. Aβ_1-42_, Amyloid-β_1-42_; Tau, total tau protein; Ptau, phosphorylated-tau; P values were obtained from Multiple Linear Regression analysis that considered age, gender, clinical ratings, and ApoE ε4 allele as covariates.

In this present study, rs2718058 was significantly associated with baseline CSF tau level (P = 0.026, S3 Table) in the overall group in one-way analysis of variance (ANOVA), and this difference remained significant when considered age, gender and *APOE* ε4 allele in multiple linear regression analysis (*P* = 0.011): AA and GA genotype showed higher tau levels when compared to GG group (Fig. 1). In addition, there were no significant relationships between *NME8* genotype and CSF Aβ _1-42_, p-Tau in the overall group (S3 Table) or the three subgroups (AD, MCI and NC).

### Neuroimaging markers analysis

**Figure 2:**
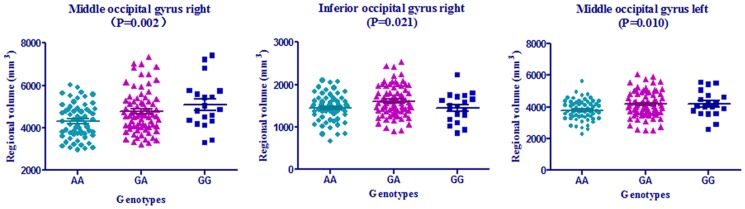
Significant brain locations and volume values on MRI at baseline in AD group. AD, Alzheimer's disease; P value is by one-way analysis of variance (ANOVA). When adjusted for age, gender and APOE ε4 allele status, P value were 0.002, 0.869, 0.051 separately.

In our analysis of 93 regions of interest regional volume via MRI in whole group and subgroups, *NME8* gene rs2718058 was significantly associated with baseline right middle occipital gyrus (*P* = 0.002), right inferior occipital gyrus (*P* = 0.021) and left middle occipital gyrus (*P* = 0.010) in the AD subgroup by the ANOVA analysis (Fig. 2). After adjusting age, gender and *APOE* ε4, the multiple linear regression analysis only identified the right middle occipital gyrus was only significantly different among the AD group (*P* = 0.002), while there was a trend for a relationship between baseline left middle occipital gyrus volume and rs2718058 in the AD group (*P* = 0.051). Additionally, compared to the AA and GA groups, the GG genotype group had larger volume in the right middle occipital gyrus in the AD participants (S4 Table, Fig. 2). We did not find a significant interaction between rs2718058 and other regions of interest in the sample.

**Figure 3:**
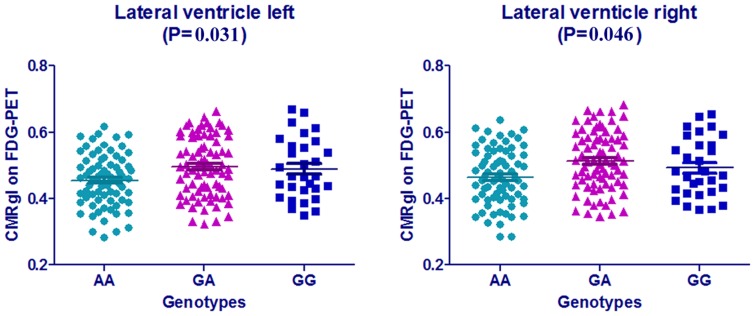
Significant brain locations and CMRgl values on PDG-PET at baseline in MCI group. MCI, mild cognitive impairment; CMRgl, cerebral metabolic rate for glucose; P values were from Multiple Linear Regression analysis that considered age, gender, and ApoE ε4 allele as covariates.

Examining the correlation between *NME8* genotype and regional brain hypometabolism via PET CMRgl across ADNI participants, the results of MCI group exhibited several positive regions, including inferior temporal gyrus, Lateral ventricle, medial frontal gyrus, corpus callosum and et al (S5 Table) in ANOVA analysis. Nevertheless, when we adjusted the age, gender and *APOE* ε4 allele status, we only identified a significant relationship between the GG genotype and higher CMRgl in both the right (P = 0.046) and left lateral ventricle (P = 0.031) in the MCI group (Fig. 3). There were no significant associations between *NME8* genotype and CMRgl in the AD, NC and whole groups.

**Figure 4:**
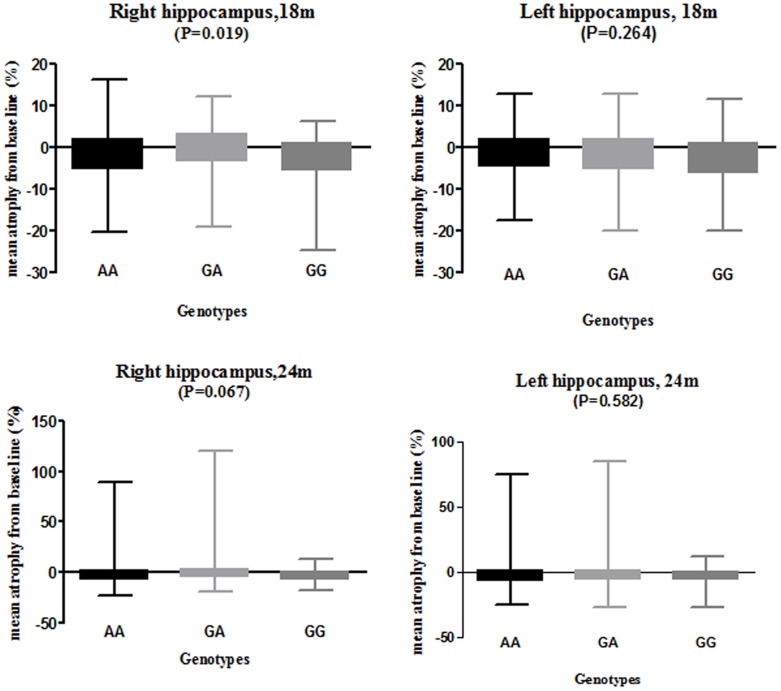
Percentage of hippocampal atrophy on MRI over 18 months and 24 months in whole group. m, month; P values were from one-way analysis of variance (ANOVA)

To further study the *NME8* rs2718058 genotype effects, we performed the analysis of percentage of hippocampal atrophy from baseline, including the left and right hippocampus atrophy, over three years (five phases: 6 months, 12 months, 18months, 24 months, 36 months) and compared the statistical power. The percentage of right hippocampus atrophy from baseline was significantly different among the three genotypes (P = 0.019) in the 18 months phase, while the right hippocampal atrophy showed marginal different among the three genotypes in the 24 months phase (P = 0.067) (Fig. 4, S6 Table).

### Statistic power

One way ANOVA power analysis by PASS 11.0 showed powerful results for our positive analyses to detect differences among the means versus the alternative of equal means using an F test with a 0.05 significance level. In the CDRSB score analysis, our NC sample achieves 100% power; in the MRI occipital analysis, our sample achieves 100% power; in the CMRgl analysis, our MCI sample achieves 93% power; in the 18 months right hippocampal follow-up analysis, our sample achieves 73% power.

## Discussion

This study provides a comprehensive evaluation of the impact of *NME8* SNP rs2718058 on cognition, 93 brain area neurodegeneration, brain metabolism and CSF Aβ, tau, and p-tau levels in HC, AD and MCI. To date, no studies have investigated the relationship of *NME8* SNP rs2718058 to AD endophenotypes in the ADNI sample. The involvement of *NME8* in neurodegenerative diseases with mental deficiency such as AD has not been reported neither. Findings from this large multi-aspect study confirmed the newly characterized protective role of SNP rs2718058 in *NME8* gene in the large meta-analysis [9].

*NME8* (NM23 family member 8), a member of NM23 family, is known to be responsible for primary ciliary dyskinesia type 6 [14]. Although the relationship between *NME8* and neurodegenerative diseases had not been well reported, proteins encoded by its family members had been reported to be associated with neurodegenerative disease in two papers [15], [16]. In brain, nucleoside diphosphate kinase (NDPK), encoded by NM23 family, have been implicated to modulate neuronal cell proliferation, differentiation, and neurite outgrowth [15], [17], [18]. Although the role of NDPK in neurodegenerative diseases has not been fully reported yet, a group from Austria found that the protein expression levels of NDPK in AD were moderately decreased, and the decreased activity of NDPK could influence neuronal processes such as neurite outgrowth and axonal sprouting by altering the expression of cytoskeletal proteins, which would in turn lead to the aberrant proliferation and differentiation of brain cells. Based on the fact that the specific activity of NDPK in brain was higher than in other tissues [19], and its expression was accumulating preferentially in the nervous system during early embryonic development [20], NDPK were predicted to play a pivotal role in the brain functions. Besides, nm23 nucleoside diphosphate kinase/metastatic inhibitory protein (PuF), encoded by nm34-H2 gene, may regulate the promoter of Aβ precursor protein gene (a gene that account for a part of familial AD) and that AD risk may be increased by interference with PuF regulation at the proximal regulatory element (PRE) [16]. All these potential connection between the NM23 family and AD may help to understand the underlying role of *NME8* in AD.

The memory decline and dysfunction are important manifestations in AD, which can be assessed by the declined cognitive function on various neuropsychological tests. Our cognitive research identified that the Clinical Dementia Rating scale sum of boxes (CDRsb) scores were different among the three genotypes in normal participants, and the difference was stronger in the 2-years later, with GG allele had lower cognitive decline. Therefore, it is possible that the *NME8* rs2718058 could improve the cognition in the general population, or even preclinical stage of AD and then delay the occurrence of AD. Nevertheless, the mechanisms of the *NME8* effect on cognition need a long time longitudinal studies to study the change of diagnosis status further.

Analysis of AD-related biomarkers in CSF (Aβ_1-42_, pTau181, and Tau) of AD, MCI and normal control groups confirmed previous findings that decreased Aβ_1-42_ and increased Tau and pTau181 which are associated with MCI and AD [21]–[24]. In our analysis, the decreased Tau was observed in the GG genotype participant in the AD group, indicating that the minor allele G may have potential role to moderate the pathology of AD. It might also be possible that this result is related to the stage in the progression of the underlying neuropathology in AD population, and this finding support the potential protective role of *NME8* in the total participants.

We found that there was significant relationship between *NME8* (rs2718058) SNP and percent of right hippocampal atrophy over 18 months in the whole groups, and right middle occipital gyrus atrophy in baseline AD group. In our analysis, the GA group was associated with lower rates of atrophy in the right hippocampus over time, while the GG group (minor-allele homozygote group) had higher rates of atrophy in the right hippocampus, an AD-related marker of neurodegeneration; GG genotype group had lower right occipital gyrus atrophy than the GA and AA groups in AD participants. Since the rate of hippocampal atrophy had been used to predict MCI to probable AD conversion, the results may suggest that the heterozygous has the potential to prevent the conversion of MCI to AD. Former studies have found the occipital lobe atrophy in dementia with Lewy bodies (DLB) [25], [26] and posterior cortical atrophy (PCA), the atypical variants of early onset Alzheimer's disease (EOAD) [27]. However, the influences of occipital atrophy in AD patients have not been fully discussed. This contradictory impact on the regional atrophy of the hippocampus and middle occipital gyrus still need to be explored further. The elevated lateral ventricle metabolism, in which atrophy features had been regarded as to be a potential biomarker to discriminating among AD, FTD and NC [28], may direct to the potential protect role of the *NME8* in AD progression. While the biological mechanisms underlying the elevated lateral ventricle CMRgl are unclear and the detailed function of the hypometabolism hadn't be revealed in the former papers, the potential role of *NME8* in brain metabolism need to be further discussed.

The strengths of ADNI database lie in its large sample size, detailed cognitive assessment protocol and careful diagnostic ascertainments, as well as in the detailed MRI, PET and CSF information and processing strategies across multiple sites and meticulous data quality control. However, the present study has few notable limitations. First, we did not evaluate all known biomarkers of AD, including FDG PET or advanced MRI techniques, which are available in subsets of the ADNI-Go/2 cohort (ADNI-Go/2 didn't provide rs2718058 information). Second, brain imaging measures of cognitive task related functional changes (by functional-MRI) would be more useful in characterizing the relationship between *NME8* and AD during memory-related tasks. Voxel-based approach, which may be useful for future studies, will characterize the regional relationship more direct. Third, our sample was restricted to Caucasians to avoid genetics stratification across ethnicities, while *NME8* (rs2718058) showed different frequencies and polymorphisms in different population. Four, not all subjects have information for all of the measurements, and the subdivisions to NC/MCI/AD subgroups and different genotypes make effective sample sizes further small for some tests. Thus, replications in other populations are imperative. Five, a follow-up of two years may be too short to detect the significant influence of *NME8* on AD. Finally, because of the limited paper to be referred, it is hard to provide a good deal of explanations for our findings. Thus, more studies are required to identify the genetic mechanisms of *NME8* (rs2718058) in the progression of AD in further studies.

In summary, here we show an association between *NME8* rs2718058 and several biomarkers of AD, which were consistent with direction of previous research. These data revealed the participation of *NME8* (rs2718058) genetic variants in delayed cognitive decline, the elevated tau levels in CSF, the hippocampus atrophy, occipital gyrus atrophy, lateral ventricle hypometabolism throughout the AD physiopathological process. Though several limitations hampered the explanation of our findings, further neuropathological and biological studies of this gene and loci may fully dissect out its role in AD. While this study is relatively large for a brain imaging and genetics report, further research in large independent samples with diverse ethnicity is required to confirm the effects of *NME8* on AD.

## Supporting Information

S1 Table Cognition scores on various neuropsychological scales in subjects at baseline.
(DOCX)

S2 Table The 2-year later cognition scores on various neuropsychological scales.
(DOCX)

S3 Table Cerebrospinal fluid (CSF) biomarkers concentration in subjects.
(DOCX)

S4 Table Significant results from analysis of MRI regions of interest with rs2718058 in AD group.
(DOCX)

S5 Table Significant interest of areas in the one-way analysis (ANOVA).
(DOCX)

S6 Table Percentage of regional hippocampal atrophy on MRI during the five phases.
(DOCX)
