# Supplementary material for: Association between NME8 Locus Polymorphism and Cognitive Decline, Cerebrospinal Fluid and Neuroimaging Biomarkers in Alzheimer's Disease
Source: PLoS One. 2014 Dec 8;9(12):e114777. doi: 10.1371/journal.pone.0114777 (PMC4259473; doi:10.1371/journal.pone.0114777)
Supplement: S1 Table — Cognition scores on various neuropsychological scales in subjects at baseline. (DOCX) [file pone.0114777.s001.docx]

**Table** 1 Cognition scores on various neuropsychological scales in subjects at baseline

| Cognition | **AA** | | **GA** | | **GG** | | ANOVA |
| --- | --- | --- | --- | --- | --- | --- | --- |
|  | N | Mean±SD | N | Mean±SD | N | Mean±SD | P |
| **Total group** |  |  |  |  |  |  |  |
| CDRSB | 283 | 1.80±1.85 | 328 | 1.71±1.83 | 107 | 1.54±1.60 | .437 |
| ADAS11 | 283 | 11.84±6.23 | 329 | 11.28±6.11 | 107 | 11.15±6.28 | .444 |
| ADAS13 | 283 | 18.26±9.23 | 326 | 17.82±9.04 | 107 | 17.85±9.45 | .828 |
| MMSE | 283 | 26.83±2.68 | 329 | 26.72±2.70 | 107 | 26.96±2.49 | .696 |
| RAVLT | 281 | 32.08±11.23 | 328 | 32.71±11.44 | 106 | 33.85±11.44 | .388 |
| FAQ | 283 | 4.78±6.39 | 327 | 4.68±6.60 | 107 | 4.70±5.99 | .979 |
| **AD group** |  |  |  |  |  |  |  |
| CDRSB | 64 | 4.46±1.58 | 79 | 4.17±1.82 | 19 | 3.84±1.38 | .327 |
| ADAS11 | 64 | 18.77±6.26 | 79 | 17.84±5.78 | 19 | 19.01±4.99 | .561 |
| ADAS13 | 64 | 28.11±8.84 | 79 | 27.65±7.79 | 19 | 29.96±7.12 | .542 |
| MMSE | 64 | 23.32±2.05 | 79 | 23.35±2.11 | 19 | 23.21±2.01 | .964 |
| RAVLT | 63 | 23.31±6.98 | 79 | 23.46±7.15 | 19 | 23.00±7.14 | .966 |
| FAQ | 64 | 12.64±6.89 | 79 | 12.94±7.12 | 19 | 13.26±5.63 | .931 |
| **MCI group** |  |  |  |  |  |  |  |
| CDRSB | 141 | 1.59±0.85 | 152 | 1.51±0.76 | 53 | 1.71±1.02 | .306 |
| ADAS11 | 141 | 11.86±3.99 | 152 | 11.18±4.36 | 53 | 11.27±5.42 | .395 |
| ADAS13 | 141 | 18.80±6.06 | 152 | 18.04±6.38 | 53 | 18.49±7.64 | .607 |
| MMSE | 141 | 27.12±1.80 | 152 | 26.97±1.80 | 53 | 26.92±1.61 | .704 |
| RAVLT | 141 | 29.68±8.58 | 152 | 30.88±9.29 | 52 | 32.90±10.5 | .090 |
| FAQ | 141 | 3.80±4.44 | 152 | 3.31±4.117 | 53 | 4.58±4.06 | .180 |
| **NC group** |  |  |  |  |  |  |  |
| CDRSB | 78 | 0.01±0.05 | 98 | 0.05±0.15 | 35 | 0.02±0.11 | **.050** |
| ADAS11 | 78 | 6.13±2.87 | 98 | 6.14±2.85 | 35 | 6.70±3.17 | .581 |
| ADAS13 | 78 | 9.21±4.14 | 98 | 9.56±4.10 | 35 | 10.30±4.58 | .444 |
| MMSE | 78 | 29.20±0.94 | 98 | 29.05±1.06 | 35 | 29.05±0.93 | .567 |
| RAVLT | 77 | 43.67±8.89 | 97 | 43.12±9.13 | 35 | 41.17±10.17 | .407 |
| FAQ | 78 | 0.12±0.46 | 98 | 014±0.53 | 35 | 0.22±1.03 | .718 |

AD, Alzheimer’s disease; MCI, mild cognitive impairment; NC, normal cognition; N, number; SD, standard deviation; P value was from one-way analysis of variance
