# Supplementary material for: Association between NME8 Locus Polymorphism and Cognitive Decline, Cerebrospinal Fluid and Neuroimaging Biomarkers in Alzheimer's Disease
Source: PLoS One. 2014 Dec 8;9(12):e114777. doi: 10.1371/journal.pone.0114777 (PMC4259473; doi:10.1371/journal.pone.0114777)
Supplement: S2 Table — The 2-year later cognition scores on various neuropsychological scales. (DOCX) [file pone.0114777.s002.docx]

**Table** 2 The 2-year later cognition scores on various neuropsychological scales

| Cognition | AA | | GA | | GG | | ANOVA |
| --- | --- | --- | --- | --- | --- | --- | --- |
| Scale | N | Mean±SD | N | Mean±SD | N | Mean±SD | P |
| **Total group** |  |  |  |  |  |  |  |
| CDRSB | 283 | 1.82±1.92 | 328 | 1.84±2.09 | 107 | 1.61±1.65 | .566 |
| ADAS11 | 282 | 11.90±6.42 | 329 | 11.49±6.95 | 107 | 11.48±6.65 | .730 |
| ADAS13 | 278 | 18.52±9.25 | 326 | 18.17±9.75 | 107 | 18.27±9.73 | .905 |
| MMSE | 282 | 26.74±2.80 | 329 | 26.59±3.01 | 107 | 26.75±2.98 | .785 |
| RAVLT | 282 | 32.11±11.65 | 328 | 32,39±33.66 | 106 | 33.66±11.81 | .499 |
| FAQ | 283 | 4.95±6.54 | 327 | 4.96±6.87 | 107 | 4.91±6.36 | .997 |
| **AD group** |  |  |  |  |  |  |  |
| CDRSB | 64 | 4.53±1.61 | 79 | 4.51±2.41 | 19 | 3.92±1.46 | .482 |
| ADAS11 | 64 | 18.987.90 | 79 | 18.89±7.9 | 19 | 19.00±5.00 | .996 |
| ADAS13 | 62 | 29.16±7.7 | 77 | 29.12±9.32 | 19 | 29.89±7.24 | .936 |
| MMSE | 64 | 23.12±2.14 | 79 | 22.89±2.92 | 19 | 23.47±2.29 | .657 |
| RAVLT | 63 | 22.87±7.28 | 79 | 23.18±7.39 | 19 | 22.26±6.49 | .877 |
| FAQ | 64 | 13.14±6.83 | 79 | 13.46±7.51 | 19 | 13.46±7.51 | .962 |
| **MCI group** |  |  |  |  |  |  |  |
| CDRSB | 141 | 1.61±1.01 | 152 | 1.58±0.81 | 53 | 1.8113±1.10 | .310 |
| ADAS11 | 140 | 11.85±4.17 | 152 | 11.19±4.44 | 53 | 11.84±6.26 | .435 |
| ADAS13 | 138 | 18.99±6.04 | 151 | 18.31±6.28 | 53 | 19.17±8.36 | .587 |
| MMSE | 140 | 27.05±1.97 | 152 | 26.96±1.87 | 53 | 26.49±2.91 | .246 |
| RAVLT | 141 | 29.62±9.08 | 152 | 30.34±9.61 | 52 | 32.50±10.84 | .181 |
| FAQ | 141 | 3.91±4.51 | 150 | 3.65±4.28 | 53 | 5..02±5.49 | .174 |
| **NC group** |  |  |  |  |  |  |  |
| CDRSB | 78 | 0±0 | 97 | 0.05±0.15 | 35 | 0.05±0.20 | **.021** |
| ADAS11 | 78 | 6.18±3.00 | 98 | 6.01±2.75 | 35 | 6.87±3.31 | .335 |
| ADAS13 | 78 | 9.25±4.19 | 98 | 9.37±4.03 | 35 | 10.61±4.84 | .256 |
| MMSE | 78 | 29.17±0.94 | 98 | 29.01±1.06 | 35 | 28.94±0.97 | .452 |
| RAVLT | 78 | 44.09±8.71 | 97 | 43.09±9.17 | 35 | 41.60±9.65 | .399 |
| FAQ | 78 | 0.11±0.45 | 98 | 0.13±0.53 | 35 | 0.22±1.03 | .653 |

AD, Alzheimer’s disease; MCI, mild cognitive impairment; NC, normal cognition; N, number; SD, standard deviation; P value was from one-way analysis of variance
