# Supplementary material for: Association between NME8 Locus Polymorphism and Cognitive Decline, Cerebrospinal Fluid and Neuroimaging Biomarkers in Alzheimer's Disease
Source: PLoS One. 2014 Dec 8;9(12):e114777. doi: 10.1371/journal.pone.0114777 (PMC4259473; doi:10.1371/journal.pone.0114777)
Supplement: S3 Table — Cerebrospinal fluid (CSF) biomarkers concentration in subjects. (DOCX) [file pone.0114777.s003.docx]

**Table** 3 Cerebrospinal fluid (CSF) biomarkers concentration in subjects

| **Biomarker** | GG | | GA | | AA | | ANOVA | Linear |
| --- | --- | --- | --- | --- | --- | --- | --- | --- |
|  | N | Mean±SD | N | Mean±SD | N | Mean±SD | *P* value | *P* value |
| **Total group** |  |  |  |  |  |  |  |  |
| Aβ_42_ (pg/ml) | 55 | 173.44±63.49 | 171 | 174.95±56.15 | 146 | 165.06±56.79 | 0.294 | 0.102 |
| Tau (pg/ml) | 55 | 86.07±45.51 | 171 | 91.45±58.59 | 146 | 105.97±57.71 | **0.026** | **0.011** |
| P-tau_181p_(pg/ml) | 55 | 30.93±19.09 | 171 | 33.09±18.99 | 146 | 35.12±18.00 | 0.318 | 0.099 |
| **AD group** |  |  |  |  |  |  |  |  |
| Aβ_42_ (pg/ml) | 10 | 141.00±45.16 | 43 | 141.63±34.09 | 35 | 147.29±47.00 | 0.810 | NS |
| Tau (pg/ml) | 10 | 106.40±40.91 | 43 | 112.16±56.16 | 35 | 136.14±65.56 | 0.148 | NS |
| P-tau_181p_(pg/ml) | 10 | 38.80±20.12 | 43 | 42.70±19.97 | 35 | 40.91±19.17 | 0.828 | NS |
| **MCI group** |  |  |  |  |  |  |  |  |
| Aβ_42_ (pg/ml) | 26 | 144.35±56.73 | 75 | 171.31±55.81 | 75 | 159.73±53.99 | 0.089 | NS |
| Tau (pg/ml) | 26 | 97.54±52.47 | 75 | 96.89±70.81 | 75 | 106.60±55.06 | 0.601 | NS |
| P-tau_181p_(pg/ml) | 26 | 37.00±16.94 | 75 | 33.60±18.89 | 75 | 36.36±17.45 | 0.561 | NS |
| **NC group** |  |  |  |  |  |  |  |  |
| Aβ_42_ (pg/ml) | 19 | 230.32±37.15 | 53 | 207.13±54.23 | 36 | 193.44±62.03 | 0.063 | NS |
| Tau (pg/ml) | 19 | 59.68±19.94 | 53 | 66.94±23.05 | 36 | 75.33±36.60 | 0.126 | NS |
| P-tau_181p_(pg/ml) | 19 | 18.47±4.89 | 53 | 24.57±14.11 | 36 | 26.89±15.33 | 0.091 | NS |

AD, Alzheimer’s disease; MCI, mild cognitive impairment; NC, normal cognition; N, number; SD, standard deviation; ANOVA, one-way analysis of variance; linear, Multiple Linear Regression analysis that considered age, gender, and APOE ε4 allele as covariates; NS, not significant; Bolded scores indicate p-values < 0.05.
