# Supplementary material for: Association between NME8 Locus Polymorphism and Cognitive Decline, Cerebrospinal Fluid and Neuroimaging Biomarkers in Alzheimer's Disease
Source: PLoS One. 2014 Dec 8;9(12):e114777. doi: 10.1371/journal.pone.0114777 (PMC4259473; doi:10.1371/journal.pone.0114777)
Supplement: S4 Table — Significant results from analysis of MRI regions of interest with rs2718058 in AD group. (DOCX) [file pone.0114777.s004.docx]

| Region | GG (mm^3^) | | GA (mm^3^) | | AA (mm^3^) | | ANOVA | Linear |
| --- | --- | --- | --- | --- | --- | --- | --- | --- |
|  | N | Mean±SD | N | Mean±SD | N | Mean±SD | P | P |
| middle occipital gyrus right | 19 | 5102.11±1151.29 | 75 | 4752.00±973.31 | 62 | 4308.08±810.54 | **0.002** | **0.002** |
| inferior occipital gyrus right | 19 | 1449.84±343.36 | 75 | 1606.45±356.35 | 62 | 1454.45±317.09 | **0.021** | 0.864 |
| middle occipital gyrus left | 19 | 4180.37±825.20 | 75 | 4164.88±806.49 | 62 | 3790.56±633 | **0.010** | 0.051 |

**Table** 4 Significant results from analysis of MRI regions of interest with rs2718058 in AD group
