# Supplementary material for: Association between NME8 Locus Polymorphism and Cognitive Decline, Cerebrospinal Fluid and Neuroimaging Biomarkers in Alzheimer's Disease
Source: PLoS One. 2014 Dec 8;9(12):e114777. doi: 10.1371/journal.pone.0114777 (PMC4259473; doi:10.1371/journal.pone.0114777)
Supplement: S5 Table — Significant interest of areas in the one-way analysis (ANOVA). (DOCX) [file pone.0114777.s005.docx]

**Table** 5 Significant interest of areas in the one-way analysis (ANOVA)

| Region | *P* (ANOVA) | *P* (Linear) |
| --- | --- | --- |
| Lateral ventricle left | 0.017 | 0.031 |
| lateral ventricle right | 0.005 | 0.046 |
| superior frontal gyrus right | 0.027 | NS |
| medial frontal gyrus left | 0.014 | NS |
| temporal pole right | 0.007 | NS |
| middle frontal gyrus left | 0.001 | NS |
| superior frontal gyrus left | 0.003 | NS |
| inferior frontal gyrus right | 0.042 | NS |
| perirhinal cortex right | 0.025 | NS |
| inferior temporal gyrus left | 0.011 | NS |
| temporal pole left | 0.024 | NS |
| corpus callosum | 0.027 | NS |
| angular gyrus left | 0.027 | NS |

NS, not significant
