# Supplementary material for: Association between NME8 Locus Polymorphism and Cognitive Decline, Cerebrospinal Fluid and Neuroimaging Biomarkers in Alzheimer's Disease
Source: PLoS One. 2014 Dec 8;9(12):e114777. doi: 10.1371/journal.pone.0114777 (PMC4259473; doi:10.1371/journal.pone.0114777)
Supplement: S6 Table — Percentage of regional hippocampal atrophy on MRI during the five phases. (DOCX) [file pone.0114777.s006.docx]

**Table** 6 Percentage of regional hippocampal atrophy on MRI during the five phases.

| Phase/location | AA | | GA | | GG | | P value |
| --- | --- | --- | --- | --- | --- | --- | --- |
|  | N | mean±SD(%) | N | mean±SD(%) | N | mean±SD(%) |  |
| **Phase1-m06** |  |  |  |  |  |  |  |
| Right hippocampal formation | 266 | 1.56±4.97 | 310 | 2.21±5.06 | 97 | 1.50±5.36 | 0.235 |
| Left hippocampal formation | 266 | 1.36±5.11 | 310 | 1.28±4.91 | 97 | 2.21±5.35 | 0.267 |
| **Phase2-m12** |  |  |  |  |  |  |  |
| Right hippocampal formation | 251 | -0.17±5.14 | 292 | 0.52±5.71 | 85 | -0.19±5.59 | 0.279 |
| Left hippocampal formation | 251 | 0.02±5.29 | 292 | -0.08±5.46 | 85 | 0.15±6.46 | 0.939 |
| **Phase3-m18** |  |  |  |  |  |  |  |
| Right hippocampal formation | 109 | -2.07±5.93 | 117 | -0.47±5.91 | 36 | -3.32±5.68 | **0.019** |
| Left hippocampal formation | 109 | -1.49±4.98 | 117 | -1.59±6.03 | 36 | -3.25±7.33 | 0.264 |
| **Phase4-m24** |  |  |  |  |  |  |  |
| Right hippocampal formation | 189 | -3.12±9.75 | 202 | -0.87±11.79 | 63 | -3.24±6.29 | 0.067 |
| Left hippocampal formation | 189 | -2.89±9.32 | 202 | -2.19±10.32 | 63 | -3.50±7.23 | 0.582 |
| **Phase5-m36** |  |  |  |  |  |  |  |
| Right hippocampal formation | 14 | -3.90±11.88 | 26 | -0.95±13.34 | 9 | -3.91±8.79 | 0.702 |
| Left hippocampal formation | 14 | -5.76±5.99 | 26 | -1.27±14.01 | 9 | -7.83±11.88 | 0.278 |

m06, 6 months; m12, 12 months; m18, 18 months; m24, 24 months, m36, 36months.
